# Isothermal Amplification Methods for the Detection of Nucleic Acids in Microfluidic Devices

**DOI:** 10.3390/bios3010018

**Published:** 2012-12-27

**Authors:** Laura Maria Zanoli, Giuseppe Spoto

**Affiliations:** 1 Istituto Biostrutture e Bioimmagini, CNR, Viale A. Doria 6, Catania, Italy; E-Mail: lzanoli@unict.it; 2 Dipartimento di Scienze Chimiche, Università di Catania, Viale Andrea Doria 6, I-95125 Catania, Italy

**Keywords:** microfluidics, isothermal amplification methods, miniaturization, DNA

## Abstract

Diagnostic tools for biomolecular detection need to fulfill specific requirements in terms of sensitivity, selectivity and high-throughput in order to widen their applicability and to minimize the cost of the assay. The nucleic acid amplification is a key step in DNA detection assays. It contributes to improving the assay sensitivity by enabling the detection of a limited number of target molecules. The use of microfluidic devices to miniaturize amplification protocols reduces the required sample volume and the analysis times and offers new possibilities for the process automation and integration in one single device. The vast majority of miniaturized systems for nucleic acid analysis exploit the polymerase chain reaction (PCR) amplification method, which requires repeated cycles of three or two temperature-dependent steps during the amplification of the nucleic acid target sequence. In contrast, low temperature isothermal amplification methods have no need for thermal cycling thus requiring simplified microfluidic device features. Here, the use of miniaturized analysis systems using isothermal amplification reactions for the nucleic acid amplification will be discussed.

## 1. Introduction

A great effort has been devoted, during the past decade, to the development of new devices for specific DNA sequences detection, due to the relevance of nucleic acid analysis in many areas, including clinical diagnostics [1], environmental monitoring and food-quality control [2,3]. However, diagnostic tools used to detect nucleic acids need to meet specific requirements in terms of sensitivity, selectivity and high-throughput in order to widen their applicability and to minimize the cost of the whole detection assay [4]. Often, a limited amount of DNA is available, particularly when biological material from human samples are going to be analyzed. This is true when, for instance, circulating cell-free fetal DNA in maternal blood or mutated DNA in rare circulating tumor cells are going to be considered. Similar limitations are also found in forensic applications due to the small amount of biological material that may be recovered from some crime scene samples. In this perspective, efforts have been paid in developing even more sensitive methods for nucleic acid detection, capable of directly transducing the hybridization events from limited biological samples [4,5,6].

It is for this reason that a key step in nucleic acid detection protocols is the amplification of the target sequence. The amplification generates a large number of target copies, greatly enhancing the assay sensitivity [7]. The most widely adopted amplification procedures exploit the polymerase chain reaction (PCR) based on a polymerase activity for primer-directed target amplification. The success of PCR amplification is related to its simplicity and its cost-effectiveness. Moreover, it has the potential for a single DNA molecule amplification (*i.e*., single-molecule PCR) [8,9] due to its highly efficient exponential process. However, despite its many advantages, PCR suffers from drawbacks which have led to the development of alternative amplification methods [7,10]. Among them, the time consuming thermal cycling between different temperatures needed to obtain the target sequence amplification. When compared to PCR methods, isothermal amplification methods, not requiring any thermal cycling, are easier to operate and require less energy than PCR methods that instead require rapid heating and cooling steps. These features greatly simplify the isothermal amplification implementation in point-of-care diagnostic devices. In addition, the use of fully enclosed microstructured devices into which performing the isothermal amplification reduces the risk of sample contamination and implies low sample consumption, multiplex DNA analysis, integration and portable devices realization. 

This review will first discuss the use of microfluidic-based systems for nucleic acid amplification by underlining advantages coming from the rapidity of the process and the low sample consumption and discussing drawbacks caused by surface-dependent processes. Miniaturized isothermal amplification systems will be then illustrated including nucleic acid sequence-based amplification (NASBA), loop-mediated isothermal amplification (LAMP), helicase-dependent amplification (HDA), rolling circle amplification (RCA), multiple displacement amplification (MDA) and recombinase polymerase amplification (RPA).

## 2. Microfluidics for Nucleic Acids Amplification

Since its advent, microfluidic has drawn great attention for the capability of miniaturizing typical laboratory operations by using a fraction of the volume of reagents in significantly less time [11]. Compared with the macroscale, the microfluidic environment offers important advantages in biochemical detection, such as shorter times for the analysis, faster mass and thermal transfer, reduced sample volumes, and potential for automation and integration. Moreover, the microfluidic environment offers fundamentally new capabilities for fluids control. 

A microfluidic device may include several components such as valves, mixers and pumps capable of controlling the fluid flow within the device and other components useful for sample pre-treatments or detection. Liquids confined into the microfluidic environment experience an interplay of multiple physical effects that lead to variants of physical properties of fluids moving in large channels. Microfluidics has been able to exploit differences existing between fluids flowing at the macroscale or travelling through microchannels, allowing one to perform techniques and experiments which are impossible at the macroscale [12]. 

In the typical low Reynolds number regime of microfluidics, viscous forces overwhelm inertial forces and fluids adopt a laminar flow [13,14]. This fact has important implications and constitutes the basis of many microfluidics-based technologies. Fluid mixing in the microfluidic regime occurs through the diffusion, thus resulting in prolonged mixing times. Purely diffusive mixing can be desirable for some applications while it is to be avoided in others. Thus, microfluidic chemical reactors require different approaches in order to bring fluids together and to mix them rapidly. The diffusion length, and hence, the mixing time can be drastically shortened by applying multiple laminar flow sheets so that millisecond time resolutions can be reached [15,16]. 

Passive mixing principles are very attractive because they rely on diffusion or chaotic advection through specially-designed microchannels. They can also operated by avoiding the need of any external energy source. Active mixing schemes, on the other hand, improve the mixing performance by applying external forces to perturb sample flows and consequently to accelerate the diffusion processes. The latter configuration often leads to a complex device design [17].

Enhanced sensitivity can sometimes be achieved by increasing the reaction time before the detection as concerned in the stopped-flow technology [18]. Using a high-precision valve system, the stopped-flow format enables the monitoring of dynamic interactions of biomolecules or other macromolecules with high temporal resolution [19].

Different microfluidic-coupled PCR amplification approaches have been developed over the last few years [20,21]. These also include reverse transcription polymerase chain reaction (RT-PCR) for RNA amplification [22,23]. Microfluidic PCR enables sample manipulation and detection to be integrated into a single device and is associated with a rapid heat transfer, due to the large surface-to-volume ratio obtained in the microfluidic device. The latter property helps in significantly reducing the thermal-cycling time while increasing the amplification reaction yield as a consequence of the more uniform heat transfer to the PCR solution during the amplification process. On the other hand, the non-specific adsorption of PCR mixture components on the surface of the microfluidic channels becomes an important limitation [24]. In fact, PCR sample interaction with the surface can produce a partial inhibition of the PCR enzyme and can carryover contamination problems [20]. Silicon/glass-based PCR microchips have shown partial or total inhibition of the PCR amplification caused by the interaction between the chip surface and PCR components [25]. Surface adsorption has been proposed as the phenomenon responsible for the inhibition. In particular, it has been shown that the PCR inhibition effect is due to a non-specific sequestering of Taq polymerase which prevents the enzyme activity and thereby the DNA amplification. At the same time, DNA is not significantly adsorbed on the surface [26,27].

Improvement of the PCR efficiencies can be accomplished through passivation procedures [28,29], either by static coating, where the inner surface of microchips is coated with a “PCR friendly” material (e.g., silanization [30] or deposition of oxide layers [26,27]), or dynamic coating, in which specific components are added to the reaction mixture (e.g., polyethylene glycol [31], polyvinylpyrrolidone, bovine serum albumin [26,27]).

The introduction of a droplet-based technology provides a convenient way to prevent most of the above-mentioned issues since amplification reactions occur within droplets. Any interaction between the PCR sample and the surface of the microfluidic device is eliminated. Droplet-based microfluidics, unlike continuous flow systems, creates discrete fraction of water solutions by using immiscible fluids that are driven into separate microchannels via an independently controlled flow [32]. Most of the adopted methods produce droplets with diameters ranging from a few micrometers to hundreds of micrometers. Polydispersity, defined as the standard deviation of the size distribution divided by the mean droplet size, can be 1–3% while droplet volumes range from femtoliters to nanoliters [33]. Factors such as the use of surfactants, the viscosity of the immiscible phase, and the wettability of the channel walls can be used to manage the size of the formed droplets. The hydrophilicity or hydrophobicity of the microchannel surface determines which liquid phase is dispersed.

The ability to rapidly create highly uniform aqueous droplets with controlled contents enables the rapid analysis of very small quantities of reagents in a portable, automated and inexpensive format. Reagents are confined in droplets and each droplet is isolated from channel walls by the immiscible liquid. A similar configuration greatly reduce the reagent dispersion and sample contaminations thus offering fundamental advantages for successful single-molecule DNA amplification. Moreover, each droplet can be considered as an isolated microreactor and each microreactor can be potentially individually controlled and analyzed [34,35] by allowing an easier parallel processing [36], higher throughput [37] and reduced sample consumption with respect to the continuous flow microfluidic control. It is also possible to split the single droplet into two or more droplets or keeping reagents separate until the proper conditions are available and then performing a controlled coalescence of droplets. Both droplet fission and fusion enhance the experimental capabilities of the droplet-based microfluidic approach.

Droplets help in achieving a rapid mixing of reagents: the rate of DNA hybridization in a homogeneous solution is about 40-fold faster than the hybridization rate in solid-liquid interfaces such as those of microarrays. 

The typical experimental configuration of devices performing droplet microfluidic PCR amplification includes an oil stream that cyclically drives droplets through alternating temperature zones for denaturation and annealing thus obtaining a rapid and efficient PCR thermocycling [38]. The implementation of PCR amplification in droplet-based microfluidic systems [39] benefits from the reduced reaction volumes that result in a decreased reagent and sample consumption. The possibility of lowering the sample requirement is particularly important when limited template material is available for the analysis [37]. In addition, the use of droplet-based microfluidic systems for PCR enables the amplification and the analysis of individual target sequences to be performed in a significantly lower time as a consequence of a higher thermal cycling speed.

A number of different isothermal amplification procedures have been introduced over the last 10 years. These include nucleic acid sequence-based amplification (NASBA), loop-mediated amplification (LAMP), helicase-dependent amplification (HDA), rolling circle amplification (RCA), recombinase polymerase amplification (RPA) and multiple displacement amplification (MDA) [7]. Isothermal methods differ from PCR in that there is no need for temperature cycling or rapid heating and cooling mechanisms as in miniaturized PCR systems.

Many miniaturized isothermal amplification systems exploit the strand-displacement activity of a DNA polymerase to cyclically amplify a target in less than an hour. Reactions take place in a microchamber or in a microchannel; reservoirs can be included to inject reagents necessary for reactions and to mix them with the sample. Primers, enzymes and reagents can also be stored in the chip and, in order to obtain multiplex analyses, different primers for several targets can be employed.

## 3. Isothermal Amplification Methods

### 3.1. Loop-Mediated Isothermal Amplification (LAMP)

Loop-mediated isothermal amplification (LAMP), described for the first time by Notomi *et al.* in 2000 [40], utilizes two sets of specially designed primers, termed inner and outer primers, and a DNA polymerase with strand displacement activity. LAMP recognizes the target sequence and generates a large amount of amplified product within 1 h. It has been widely applied for the detection of pathogens such as human immunodeficiency virus (HIV) [41], severe acute respiratory syndrome coronavirus (SARS-CoV) [42], Staphylococcus aureus [43], Salmonella enterica [44].

**Figure 1:**
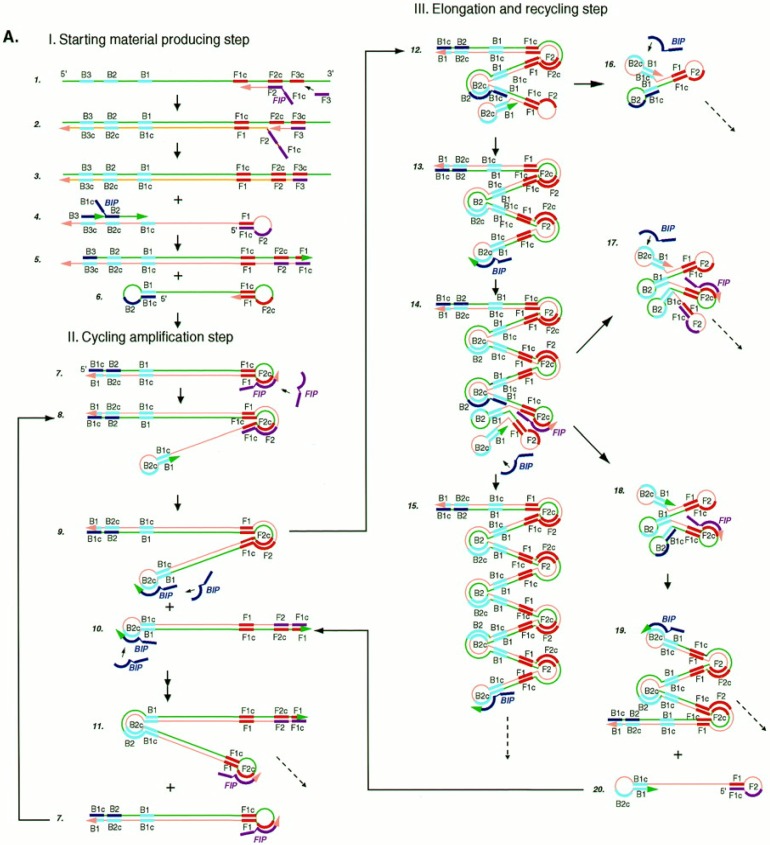
Schematic representation of the loop-mediated isothermal amplification (LAMP) amplification process. Adapted with permission from [40].

LAMP reaction is initiated by a forward inner primer (FIP), containing sequences of the sense strand of the target DNA, which hybridizes to F2c in the target and initiates complementary strand synthesis (Figure 1). Then, the outer primer F3 hybridizes to F3c portion in the target sequence leading to the displacement of the just synthesized strand that is release as a single-stranded DNA with a loop out structure at one end. The FIP-linked complementary strand acts as the template for a new DNA synthesis primed by backward inner (BIP) and outer (B3) primers that hybridize to the other end of the target, leading to the production of a dumb-bell form DNA which produces a stem–loop DNA structure as a consequence of self-primed DNA synthesis.

The subsequent cycles, comprising elongation and recycling steps, lead to final products constituted by a mixture of stem-loop DNAs having various stem lengths and cauliflower-like structures with multiple loops formed after the annealing between alternately inverted repeats of the target sequence in the same strand. It is to underline that only inner primers are used for strand displacement DNA synthesis during cycling reactions while all four primers are used during initial steps of the LAMP reaction. The initial use of four primers that enables the recognition of six distinct sequences, followed by the use of two primers, ensures the high selectivity for target amplification.

The LAMP method does not require thermocycling since the amplification is performed at a constant temperature between 60 °C and 65 °C. Another important advantage of the LAMP method is its simplicity for detection of the amplification reaction. In fact, pyrophosphate byproducts are produced during the reaction. These byproducts form a white precipitate that increases the turbidity of the solution. The detection of amplification products can be obtained by using a variety of methods including electrophoretic [45], turbidimetric [46,47] and electrochemical [48] methods or by simply visually evaluating the solution color change resulting from the SYBR green stain [49]. 

Although most analytical tools rely on bulk measurements of LAMP amplification, LAMP has also been down-scaled by integrating it in microfluidic devices. Lee *et al.* have developed an integrated isothermal device for both amplification and real-time detection of hepatitis B virus (HBV) DNA via the LAMP amplification method [50]. The device was composed of a disposable polymethyl methacrylate (PMMA) micro-reactor and a temperature-regulated optical detection unit for the real-time monitoring of the turbidity changes due to magnesium pyrophosphate precipitation. An important drawback of this specific detection scheme is represented by the volume it requires (LAMP reaction volume 25 µL) [50,51]. It should be taken into account that visual detection from microchannels suffers from low sensitivity as a consequence of the short optical length. 

Zhang *et al.* presented an on-chip LAMP carried out with a total reaction volume of 10 μL for visual determination by naked eye with SYBR Green I [52]. The reaction volume has been further reduced to 5 μL by Fang *et al.*, who integrated the LAMP method in a microfluidic chip called microLAMP (μLAMP) to quantitatively detect target nucleic acids [53]. In particular, the detection of μLAMP was performed in an eight-channel chip for readout either by the naked eye or via absorbance measured by an optic sensor.

Potential for further miniaturization has been demonstrated by the successful analysis of single DNA templates encapsulated in a polyacrylamide (PAA) gel based microchamber [54]. A microheater, regulated by an automatic feedback system was used to activate the amplification reaction in order to allow fluorescent imaging of LAMP at a single molecule level.

The electrochemical detection of microfluidic integrated LAMP amplicons has been applied for the detection and the quantification of Escherichia coli [55]. Among many sensing techniques developed to monitor biorecognition, electrochemical methods provide sensitivity, selectivity, and low cost detection of the amplified DNA sequences. If compared with optical detection, the electrochemical detection shows significant advantages such as independence from sample turbidity and extremely low-cost/low-power requirements, in addition to its compatibility with miniaturization processes. Unlike other electrochemical techniques [56], based on the immobilization of an oligonucleotide probe onto an electrode, hybridization of a complementary target sequence, and transduction of the hybridization event [57], the microfluidic detection of LAMP amplicons [55] does not require probe immobilization and bacteria detection can be accomplished in a single chamber without DNA extraction and purification steps, since the used isothermal temperature (66 °C) provides enough thermal shock to lyse the *E. coli* bacterial target without any pre-treatment.

### 3.2. Helicase-Dependent Amplification (HDA)

Progresses in understanding properties of helicases, discovered in *Escherichia coli* in 1976 [58,59], have enabled researchers to combine helicases with polymerases and other accessory proteins for applications in nucleic acid amplification. Helicase-dependent amplification (HDA) exploits the activity of a DNA helicase to separate complementary strands of double strand (ds) DNAs thus avoiding the temperature cycling to produce single-stranded templates for primer hybridization and subsequent primer extension by a DNA polymerase [60]. It mimics the denaturation mechanism in living organisms where DNA is replicated by DNA polymerases with the aid of DNA helicase to separate complementary DNA strands. 

**Figure 2:**
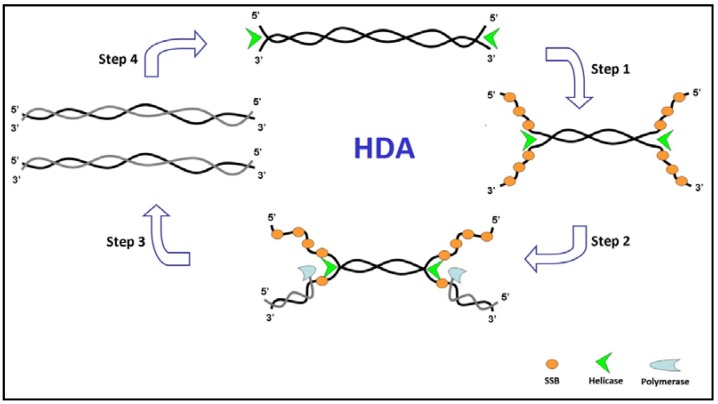
Schematic representation of helicase-dependent amplification (HDA) amplification process.

A scheme of HDA mechanism is shown in Figure 2. It mimics the replication fork and enables DNA synthesis to occur by using chemical energy [61]. The helicase enzyme in the presence of ATP loads on to the dsDNA template and traverses along the target DNA, disrupting the hydrogen bonds linking the two strands. The formed single strand (ss) DNAs are then coated by single-stranded binding proteins (SSBs; Figure 2, step 1). Two sequence-specific primers hybridize to the 3'-end of each ssDNA template (Figure 2, step 2). DNA polymerases extend primers annealed to the target by producing dsDNA (Figure 2, step 3). The two newly synthesized dsDNA products act then as substrates for DNA helicases in the next round of the reaction (Figure 2, step 4), resulting in an exponential amplification of the selected target sequence. 

Helicases are motor proteins that bind nucleic acids and move directionally along them. They separate dsDNA by using the energy generated by the nucleoside 5'-triphosphate (NTP) hydrolysis. Helicases can be classified according to their directionality (e.g., 5'–3' or 3'–5'), their source (e.g., prokaryotic or eukaryotic helicases), their oligomeric or monomeric nature and their substrate specificity (e.g., RNA *vs.* DNA) [62,63,64]. SF1 and SF2 helicase structures have shown that the DNA helicase motifs are clustered together in the tertiary structure, forming a nucleic acid-binding site and an NTP-binding pocket [62]. Conserved motifs can be envisioned as an engine that powers the unwinding of duplex nucleic acids, using both the energy derived from the nucleotide hydrolysis as well as conformational changes that allow the transduction of energy. Non-conserved portions of helicase structure, instead, are likely to encompass protein-protein interaction domains, cellular localization signals, site-specific DNA recognition domains and oligomerization interfaces, which constitute properties that are unique for each helicase [65].

The first HDA system was developed by using *Escherichia coli* UvrD helicase (~82 kDa) [60] that can operate on blunt-ended DNA fragments as well as nicked circular DNA [66]. UvrD belongs to the superfamily 1 (SF1) and shows unidirectionality in dsDNA unwinding (3'–5'). In the absence of accessory proteins (SSB. In, MutL and T4 gene 32 protein), no amplification products were produced. SSBs specifically bind to the single-stranded part of DNA in a sequence-independent manner. They enhance polymerase activity by protecting unwound ssDNAs from degradation and by destabilizing DNA secondary structure [67,68].

The limited capability of UvrD HDA system in efficiently amplifying long target sequences [69,70] was improved by using a thermostable UvrD helicase (Tte-UvrD) purified from thermophilic bacteria and the *Bacillus stearothermophilus* polymerase I at 60–65 °C [71].

Multiple reports on the use of HDA for the detection of bacterial and viral target DNA and RNA in standard microtube protocols have been described [72,73]. Recently, the use of HDA system for the detection of antibiotic-resistant pathogens *N. gonorrhoeae* and *S. aureus* directly on surface bound primers has been reported [74]. It combines HDA with a microarray-based detection, making it suitable for multiplex detection. The helicase dependent OnChip amplification (OnChip-HDA) provides a way for HDA to be developed into a miniaturized detection system for point-of-care diagnosis in conjunction with commercially available thermostable HDA kits [75].

Ramalingam *et al.* [76] described a microfluidic devices with an array of open (unsealed) reactors preloaded with primers in conjunction with a single-step capillary-based flow scheme for sequential distribution of the amplification mixture and HDA isothermal amplification of nucleic-acid. In this case, the BNI-1 fragment of SARS cDNA was successfully amplified in a real-time format at 62 °C by using the IsoAmp tHDA kit (New England Biolabs, Beverly, MA, USA) containing thermostable UvrD helicase (Tte-UvrD) and thermostable DNA polymerase. As a consequence of the limited speed and processivity of the UvrD helicase, the system was able to amplify only short DNA sequence (from 70 to 120 bp) [71].

It has been reported that the speed and the robustness of HDA are highly dependent on the synchronization of helicase, single-stranded DNA binding protein and large fragment of Bst DNA polymerase [77]. By increasing the concentration of the constituent enzymes, while maintaining the relative proportions of each type of enzyme constant, the overall reaction speed has been increased. The increasing of the primer concentration also improved the reaction speed but also produced a partial loss in amplification accuracy.

The integration in a microfluidic chip of isothermal amplification by HDA and micro solid phase extraction (μSPE) column for DNA isolation has been also described [78]. In this case, the bacteria lysis, the nucleic acid extraction and DNA amplification with a fluorescent reporter were incorporated into a disposable polymer cartridge to detect the presence of whole bacterial cells in a liquid samples. 

### 3.3. Rolling Circle Amplification (RCA)

**Figure 3:**
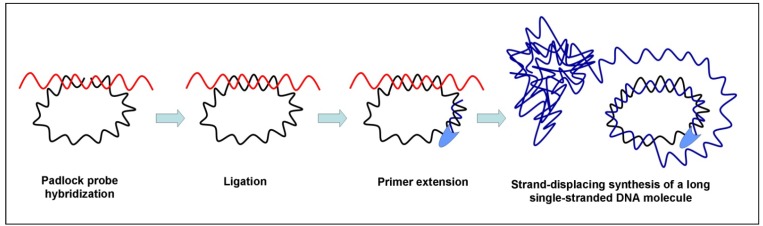
Principal mechanism for rolling circle amplification (RCA).

The rolling circle amplification (RCA) method exploits the continuous amplification of a circular DNA template by a strand displacing DNA polymerase. The DNA polymerase displaces the synthesized strand and ‘rolls’ on with DNA synthesis. This method operates at a constant temperature and produces a long single-stranded DNA molecule with tandem repeats of the circular template [79]. Both linear and exponential RCAs have been developed. In linear RCA, a small circle sequence is amplified by polymerase extension of a complementary primer, whereas in exponential RCA two primers are used: the second primer hybridizes with the single-stranded DNA product of the first primer and initiates hyper-branching in the DNA replication [80]. A schematic illustration of the principle of rolling-circle amplification is shown in Figure 3.

Efficient amplification by RCA requires small and circular single-stranded DNAs acting as the template. Unfortunately, a large number of relevant diagnostic DNA targets are composed of double stranded linear DNA molecules thus making it difficult the amplification process to be established. For this reason, oligonucleotides called padlock probes have been designed [81]. Padlock probes are oligonucleotides with the two lateral sequences complementary to two target sequences that are connected by a linker sequence. When the padlock probe hybridizes the target sequences it circularizes. Upon recognition of the target, the padlock segments are sealed through the action of a DNA ligase alone or in combination with a DNA polymerase [82]. As the circularizing step of a padlock probe is strictly target-dependent, high enough specificity is ensured to allow SNP analysis.

Sample pre-treatments are required before the padlock probe circularization in order to allow the transformation of duplex genomic DNA into accessible single-stranded DNA targets. For this reason genomic DNA is first digested with restriction enzymes that produce small sized dsDNA, then subjected to degradation by 5'-3' exonucleolysis. Even mRNA can be amplified through the RCA approach but, in this case, an mRNA pre-treatment is performed which consists of cDNA synthesis by a reverse-transcription followed by mRNA degradation by RNase H digestion [10]. In both cases, RCA of padlock probes produces a large DNA product that collapses into a submicrometer-sized concatamer that can be tagged and analyzed for single molecule detection [83]. The creation of one rolling circle product (RCP) for each recognized target makes optical detection and quantification possible by using fluorescence optical microscopy [84].

Although small circular DNA molecules are uncommon in higher organisms, they are widespread in several microorganisms such as viruses, bacteriophages and bacteria. RCA has also been used to amplify circular genomes of DNA viruses, to identify phage genomes carrying lethal mutations and bacteria by using primers that include the sequence of the target DNA [85,86]. The method is called multiply primed RCA and uses φ29 DNA polymerase and an exonuclease-resistant random primer.

Mahmoudian *et al.* introduced an integrated platform for RCA and circle-to-circle amplification (C2CA) of padlock probes on a poly(methyl methacrylate) (PMMA) microchip [87]. C2CA is an RCA-based amplification method for small DNA circles that produces single stable dsDNA fragments trough the repetition of a few (up to three) cycles of replication, monomerization, and ligation steps [88]. After each cycle of C2CA, RCA products generated in the replication step are subjected to restriction digestion to be cut into linear monomer and then subjected to ligation that produces circular ssDNA monomers. The introduction of an alternative step for ligation, termed “duplication”, enables the conversion of ssDNA to dsDNA product [89]. In the latter case, RCA and C2CA were successfully carried out in a microchip and the products were subsequently detected on the same channel of the microchip by micro-electrophoresis (µ-CE). One of the major advantages of this integrated platform is that it combines the simple RCA temperature control (stable temperature at 37 °C) with versatility, high speed, easy automation, low cost and simplicity of µ-CE, providing a reliable and fast technique for SNPs analyses.

A droplet of microfluidics-based hyperbranched rolling circle amplification (HRCA) for single-molecule DNA amplification and analysis has been also described [90]. The microfluidic devices included separate devices for droplet generation, fusion and detection and allowed the amplified DNA in each of the 2 pL droplets to be analyzed by measuring the activity of the encoded enzyme (β-galactosidase) after the fusion of the 2 pL droplet with a 15 pL droplet containing an *in vitro* translation (IVT) system supplemented with fluorescein-di-β-D-galactopyranoside (FDG) that is transformed into fluorescein by β-galactosidase.

Another RCA-based system that benefits from the typical low sample consumption and short processing time advantages of microfluidics is the rolling-circle enhanced enzyme activity detection (REEAD). REEAD was combined with a droplet microfluidic lab-on-a-chip platform by Juul *et al*. [91].

The principle of REEAD approach is the circularization of RCA substrate by the Plasmodium enzyme topoisomerase I (pTopI) together with the conversion of single DNA cleavage-ligation events to micrometer-sized products detectable at the single-molecule level. The REEAD-on-a-chip procedure appears attractive since it allows the specific, quantitative and highly sensitive detection of the human-malaria-causing Plasmodium parasites in small volumes (pL droplets) of unprocessed and crude clinical samples with a detection limit of less than one parasite/μL. 

### 3.4. Multiple Displacement Amplification (MDA)

MDA is an isothermal method for whole genome amplification (WGA) capable of producing a large number of amplification products from a few DNA molecules [92]. MDA exploits both random exonuclease-resistant primers as well as the strand displacement activity of φ29 DNA polymerase to produce DNA strands 70 kb in length [93] with no need for thermal cycling. Random primers and φ29 DNA polymerase are already used for circular DNA amplification in RCA [85,86]. In MDA the above-mentioned components are employed to amplify linear genomic DNA. The use of a high-fidelity proofreading DNA polymerase ensures a significantly lower error rate during the amplification reaction (3×10^−6^ mutations/nucleotide) [94] in comparison with Taq DNA polymerase used for PCR amplification (~2 × 10^−4^ mutations/nucleotide) [95,96]. The high-fidelity amplification by MDA preserves DNA sequence information, assuring accurate genotyping in downstream applications regardless of the type of clinical sample used including buccal swabs, whole blood, finger stick blood and Guthrie cards [97]. 

MDA does not require the temperature-modulated denaturation of the genomic DNA to facilitate the annealing of random primers. In fact, the random priming on the double-stranded DNA target is efficient enough to obtain an initial priming step that is followed by the strand-displacement process that produces the amplification. It has been also demonstrated that the omission of the denaturation step results in a reduced template degradation and in an improved specificity of the amplification [92]. MDA has been directly used on lysed cells thus avoids the DNA template loss and damage and the risk of contamination that can occur in DNA purification processes [97]. The possibility to directly apply MDA to genomic material obtained from single cells helps in eliminating the need to develop culture methods by also allowing the direct analysis of genomic DNA from nonculturable bacteria. MDA has been shown to produce several-billion-fold amplification of genomic DNA from single, flow-sorted bacterial cells [98]. Unfortunately, only about 30% of the DNA amplification was specific because MDA tends to amplify all of the DNA present in a sample, including contaminating DNAs. By using a microfluidic device comprising 60 nl reactors and an integrated cell sorter to isolate selected individual cells, it has been shown that the amplification bias can be reduced and the specificity of the amplification process can be increased as the reaction volume shrinks from microliters to nanoliters [99]. Reduced MDA reaction volumes lower nonspecific amplification that can result from contaminant DNA templates or unfavorable interactions between primers [100].

It is important to recognize that MDA tends to be a self-limiting reaction. The amplification yield is independent of the input amount of DNA template and reaches a plateau at a 0.7–1.0 µg/µL concentration of the amplified DNA [97].

### 3.5. Recombinase Polymerase Amplification (RPA)

RPA is a low temperature (about 37 °C) isothermal amplification method that couples the isothermal recombinase-driven primer targeting of the template material with the strand-displacement DNA synthesis. The method, introduced by Piepenburg *et al.* in 2006 in combination with a novel probe-based detection approach [101], amplifies DNA sequences by using a recombinase, DNA polymerase and DNA-binding proteins.

RPA uses nucleoprotein complexes constituted by oligonucleotide primers and recombinase proteins to facilitate the primer binding to the template DNA. In particular, recombinase-primer complexes scan the double-stranded DNA by promoting the primer binding at the target sequence of dsDNA and the displacement of the non-template strand. The displaced strand is stabilized by ssDNA binding proteins while the recombinase disassembly leaves 3'-end of the primer accessible to DNA polymerase. An exponential amplification of the target sequence is obtained after the cyclic replication of the process.

RPA isothermal amplification has been integrated in a centrifugal lab-on-a-chip device [102]. The fluidic cartridge of the device was fabricated with a thermoforming process and included fluidic cavities for the processing of up to 30 samples in parallel in separate 10 µL microchambers. Liquid and dry reagents for the amplification were pre-stored on the chip while the DNA sample was directly added to each microchamber in order to minimize the influence of surface adsorption effects. Spinning was used to transfer the liquid to the chamber containing the lyophilized RPA mixture and to move fluids between chambers.

Hakenberg *et al.* developed a passive microfluidic batch mixing chip for RPA fabricated through a simple and low-cost method which combines dry film resist technology and direct wafer bonding [103]. In this detection assay, based on the phaseguided fluid handling [104], fluorescence results are measured directly from the chip after a one minute mixing sequence.

Digital microfluidic isothermal amplification methods that take place at temperatures close to the room temperature are convenient because the polymerization reaction is chemistry rather that temperature actuated. However, these methods suffer from the unwanted characteristic that the amplification reaction is expected to proceed at room temperature if the nucleic acid sample is premixed with initiation reagents prior to compartmentalization. Conversely, in digital PCR the amplification reaction is controlled by temperature and any low-temperature non-specific pre-amplification process can be eliminated by using specific procedures [105]. As a consequence of that, the mixture can be compartmentalized prior to initiation with minimal risk of false positives if the infrastructure for an accurate temperature control is readily available. In point-of-care settings, a digital isothermal nucleic acid amplification approach like RPA represents an attractive option for nucleic acid quantification but, in order to achieve digital RPA without false-positive errors, the nucleic acid template must be compartmentalized prior to adding initiation reagents.

RPA has been implemented in a droplet microfluidic chip for the simultaneous chemical initiation of over one thousand sequence-specific RPA in parallel [106]. The amplification process was obtained by adding a chemical initiator to each reaction compartment with a simple slipping step after instrument-free pipet loading. The digital RPA did not require precise temperature control since it is tolerant to fluctuations of the incubation temperature ranging from 37 °C to 42 °C. The generation of amplified target material was monitored by fluorescence by using a system in which a fluorophore/quencher bearing probe was nucleolytically cut in response to sequence-specific binding to amplified DNA leading to an increase in observable fluorescence as a result of separation of the fluorophore and quencher groups.

### 3.6. Nucleic Acid Sequence-Based Amplification (NASBA)

NASBA is an isothermal, transcription-based amplification method specifically designed for the amplification of single-stranded RNA or DNA sequence. It was first introduced by Compton in 1991 [107] and is performed at 41 °C. Thanks to the integration of the reverse transcription activity into the amplification process, the method is especially suited for RNAs such as mRNA, rRNA, tmRNA or genomic RNA [108]. However, NASBA cannot amplify double-stranded DNAs not subjected to a denaturation step [109].

NASBA utilizes two RNA target-specific primers and three enzymes (*i.e*., avian myeloblastosis virus reverse transcriptase, T7 DNA-dependent RNA polymerase (DdRp) and RNase H). 

**Figure 4:**
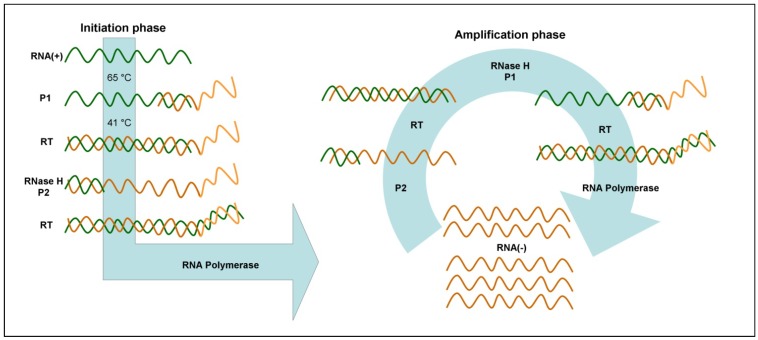
Schematic representation of nucleic acid sequence-based amplification (NASBA).

The standard NASBA protocol for RNA amplification (Figure 4) requires a 65 °C RNA incubation step to denature the target prior to the addition of enzymes. In the initiation phase, a specific forward primer (P1), that possesses a 5' sequence corresponding to the promoter of the T7 DdRp, hybridizes to any target RNA present in the sample and is extended by the reverse transcriptase. Subsequently, the RNA portion of the resulting RNA:DNA heteroduplex is degraded by RNase H, while a specific reverse primer (P2) hybridizes to the complementary sequence and is extended by the reverse transcriptase, leading to the formation of a dsDNA with the target sequence and a T7 promoter. Then, the T7 DdRp produces many RNA molecules that are complementary to the original target RNA. In the amplification phase, each newly synthesized RNA can be copied, resulting in an exponential amplification of RNA complimentary to the target.

Single-stranded RNA products are detected by using hybridization-based detection protocols with sequence-specific probes. These methods include the electrochemiluminescence [110,111], the lateral flow [112] and the electrochemical detection [113]. 

An attractive method for the detection of NASBA products exploits molecular beacons (MBs) [109] which are single-stranded nucleic acid molecules that possess an hairpin structure [114]. A fluorophore and a quencher moieties are kept in close proximity to each other due to the stem-and-loop structure, so that the fluorescence of the reporter is quenched by fluorescence resonance energy transfer (FRET) to the quencher [115]. Upon probe-target interaction, a probe conformational change occurs and the reporter is no longer in close proximity to the quencher, thus emitting a fluorescent signal. Molecular beacon NASBA fluorescence signal can be detected in real-time and is proportional to the amount of target RNA sequence [116,117].

Real-time NASBA technology has been integrated in a microfluidic system by Gulliksen *et al.* [118]. In this case, the amplification of 1.0 and 0.1 ìM oligonucleotides (human papillomavirus (HPV) 16 and artificial 118-bp single-stranded DNA) was performed in a silicon-glass microchip device comprising heated 10 nL and 50 nL reaction chambers. A surface treatment was required in order to prevent the adsorption of reaction mixture components on the surface of the microfluidic device.

An improvement in the real-time NASBA integration in microfluidic environment was introduced by detecting artificial human papilloma virus (HPV) 16 sequences and SiHa cell line samples in cyclic olefin copolymer (COC) microchips [119]. In this multipurpose microchip platform, the sample was automatically distributed into 10 parallel reaction channels for simultaneous detection in 80 nL volumes, making it possible to specifically amplify and detect several different targets with high sensitivity by only using one single sample. 

More recently Gulliksen *et al.* presented the development of a “proof of principle” self-contained and hands-free diagnostic platform for NASBA which performs sample preconcentration, nucleic acid extraction, amplification and real-time fluorescent detection with minimal user interfacing [120]. The automated platform comprises two distinct microfluidic chips, one for sample preparation and the other for nucleic acid amplification, and was applied to the detection of HPV mRNA in clinical specimens. A more complex device was obtained after the monolithic integration of a microfluidic sample-to-answer transfer-mRNA (tmRNA) analysis system that included RNA capture and purification, NASBA amplification, and real-time detection [121]. The microfluidic NASBA system was based on microchannels and microchambers, with distinct functional domains: a silica bead-bed RNA purification chamber (volume 0.25 µL), that enabled the capture of RNA by its selective adsorption on silica, and a NASBA chamber (volume 2 µL), where the amplified tmRNA target was detected in real-time via molecular beacons fluorescence detection. The integration of the sample preparation, amplification and detection compartments in a single microfluidic device reduces the risk of sample degradation, which can compromise assay accuracy, reliability and reproducibility.

A lab-on-a-chip (LOC) device based on the real-time immuno nucleic acid sequence-based amplification (immuno-NASBA) for quantitative detection of waterborne pathogens has been recently designed by Zhao *et al.* [122]. The immuno-NASBA assays relies on the use of different antibodies to recognize different bio-targets and on signal amplification by NASBA reaction before the detection. This approach enables the quantification of traces of waterborne pathogens at femtomolar levels with high specificity. One of the main advantages of the developed system is that the microfluidic device is modelled on a 96-well ELISA microplate, so it is compatible with the conventional equipment in a biological laboratory. On the other hand, it is not free from drawbacks, since it is prone to ribonuclease (RNase) contamination and non-specific adsorption that can affect the amplification signal and the signal curve of NASBA assay.

## 4. Comparison of Isothermal Amplification Methods for Microfluidic Integration

Isothermal DNA amplification is rapid, cost effective, easy-to-use and more tolerant to inhibitory components from a crude sample compared with PCR, which greatly simplifies point-of-care diagnosis, but at the same time showing equivalent or higher sensitivity and reliability in clinical diagnosis. The use of fully enclosed microstructured devices into which performing the isothermal amplification reduces the risk of sample contamination and allows integration and portable device realization. Furthermore, the implementation of isothermal DNA amplification in microfluidic systems has the added benefit of reduced reaction volumes that result in a decreased reagent and sample consumption. The possibility of lowering sample requirement constitutes an important usefulness, especially when limited template material is available. Reactions take place in a microchamber or in a microchannel; reservoirs can be included to inject reagents necessary for reactions and to mix them with the sample. Primers, enzymes and reagents can also be stored in the chip and, in order to obtain multiplex DNA analyses, different primers for several targets can be employed.

The described approaches differ from each other in terms of operating temperature, diagnostic application, mechanism, strengths and weaknesses. From this perspective, it is worthy of noting that NASBA, RCA, and RPA reactions allow nucleic acid amplification at low temperature, thus requiring less energy to operate than higher temperature isothermal amplification (*i.e*., LAMP and HDA). 

Many miniaturized isothermal systems exploit the strand-displacement activity of a DNA polymerase (LAMP, RCA, RPA, MDA) to cyclically amplify a target in less than an hour. 

Target application can vary from single stranded DNA or RNA molecules to double stranded DNA samples. DNA is often preferred, where appropriate, because it is more stable than RNA and will be likely associated with a higher sensitivity in samples stored or transported in suboptimal conditions.

A summary of isothermal nucleic acid amplification methods here described is provided in Table 1. 

**Table 1:** Summary of isothermal nucleic acid amplification methods.

Method	Amplification time	Reaction volume	Target	Detection limit ^a^	Ref.
LAMP	within 1 h	25 µL	hepatitis B virus (HBV) DNA	50 copies/25 μL	[50,51]
	within 15 min	10 µL	prostate-specific antigen gene	23 fg/μL	[52]
	within 1 h	5 µL	Pseudorabies virus (PRV) DNA	10 fg	[53]
	within 1 h	^b^	λDNA	two molecule	[54]
	1 h 35 min	35 µL	*E. coli* genomic DNA	24 colony forming units (CFU)/mLl 48 CFU/mL	[55]
HDA	2 h	150 µL	N. gonorrhoeae genomic DNA Methicillin resistant *S. aureus* genomic DNA	1 ng 250 pg	[74]
	0.5 h	~5 µL/192 nL	BNI-1 fragment of SARS cDNA	0.01 ng/μL	[76]
	0.5 h	25 µL	*E. coli* genomic DNA	10 CFU	[78]
RCA	within 65 min	10 µL	Genomic DNA for *V. cholerae*	25 ng	[87]
	4 h	2 pL	pIVEX2.2EM-lacZ plasmid	0.07 pg/μL	[90]
	2.5 h	pL	Human-malaria-causing Plasmodium parasites	less than one parasite/μL	[91]
MDA	10–16 h	60 nL	*E. coli* genomic DNA	^b^	[99]
RPA	within 20 min	10 µL	mecA gene of *Staphylococcus aureus*	less than 10 copies	[102]
	1 h	9 nL	Methicillin-resistant *Staphylococcus aureus* genomic DNA	300 copies/mL	[106]
NASBA	within 2 h	10 nL	Human papillomavirus (HPV)	1.0 ìM	[118]
	2,5 h	80 nL	Artificial human papilloma virus (HPV) 16 sequences SiHa cell line samples	10^−6^ ìM 20 cells/μL	[119]
	0,5 h	2 µL	*E. coli* tmRNA	100 cells in 100 ìL	[121]
	2–3 h	30 ìL	Water pathogens	10^5^ CFU/mL	[122]

^a^ The lowest detected concentration is shown when the detection limit is not reported; ^b^ Not available.

It should be noted that the choice of the method is strictly related to the target. NASBA for example is especially suited for RNAs such as mRNA, rRNA, tmRNA or genomic RNA, while it cannot amplify double-stranded DNAs unless subjected to a denaturation step. On the other hand, NASBA is not free from drawbacks, since it is prone to ribonuclease (RNase) contamination and non-specific adsorption that can affect the amplification signal and the signal curve of NASBA assay.

Contrary to NASBA, LAMP enables duplex DNA amplification because, in the condition of dynamic equilibrium at 65 °C, one of the LAMP primers can anneal to the complimentary sequence of double-stranded DNA without any initial denaturation step. LAMP can also be applied to the detection of RNA with the addition of reverse transcriptase. Another important advantage of the LAMP method is its simplicity for detection of the amplification reaction, due to the insoluble pyrophosphate byproduct that can be easily quantified turbimetrically.

In a different manner, the HDA reaction exploits the activity of a DNA helicase in combination with other accessory proteins to separate complementary strands of ds DNAs, thus mimicking the denaturation mechanism in living organisms. However HDA suffers from the limited speed and processivity of UvrD helicase that is able to amplify only short DNA sequences (from 70 to 120 bp). As an attempt to circumvent this drawback, the limited capability of UvrD HDA system in efficiently amplifying long target sequences was improved by using a thermostable UvrD helicase (Tte-UvrD).

The target applications of the RCA method are wide, ranging from small and circular single-stranded DNAs to double stranded linear DNA molecules and even mRNA. Sample pre-treatments are required before padlock probe circularization in order to allow the transformation of duplex genomic DNA into accessible single-stranded DNA targets; this additional heating step is responsible for increased power demand and more complicated control mechanisms. In mRNA detection, instead, the pre-treatment step consists of cDNA synthesis by a reverse-transcription followed by mRNA degradation by RNase H digestion. On the other hand, ss the circularizing step of a padlock probe is strictly target-dependent, high enough specificity is ensured to allow SNP analysis. Another relevant advantage of RCA is its capability to detect miRNA samples, which arises from its inherent nature for specific detection of short sequences. 

In RPA, an initial denaturation step is not required thanks to the activity of recombinase-primer complexes that scan the double-stranded DNA by promoting the primer binding at the target sequence and the displacement of the non-template strand. The RPA reaction has the ability to proceed at a variety of temperatures since it is tolerant to fluctuations of the incubation temperature ranging from 37 °C to 42 °C. This remarkable property of RPA is of great appeal for field applications where precise temperature control is often technically challenging.

MDA exploits random exonuclease-resistant primers and the activity of φ29 DNA polymerase, already used in RCA, but to amplify linear genomic DNA rather than for circular DNA amplification. The random priming on the double-stranded DNA target is efficient enough to obtain an initial priming step that is followed by the strand-displacement process. The MDA method is capable of producing a large number of amplification products from a few DNA molecules regardless of the type of clinical sample used including buccal swabs, whole blood, finger stick blood and Guthrie cards. In addition, the use of a high-fidelity proofreading DNA polymerase ensures a significantly lower error rate during the amplification reaction and preserves DNA sequence information, an advantage to consider when an accurate genotyping is required as in whole genome amplification (WGA).

## 5. Conclusions

The microfluidic lab-on-a-chip technology for the amplification of nucleic acids offers great potential for lower cost, higher speed, smaller sample consumption and automation of all processes from sample preparation to signal detection. The integration of multiple functional fluidic modules enables the analysis of smaller sample volumes with increased reproducibility, faster analysis and more accurate quantification.

In this review, the use of microfluidic-based methods for isothermal nucleic acid amplification has been described. In particular, the integration in microfluidic device of NASBA, LAMP, HDA, RCA, MDA and RPA isothermal amplification methods have been discussed. Isothermal reactions allow nucleic acid amplification at both constant and low temperature. Although PCR is the most widespread technology for DNA amplification, the need for an electrically powered thermal cycler with a precise temperature control and for an optimized experimental setup makes the use of PCR at point-of-care settings more complex and increases the cost of PCR-based devices. Conversely, isothermal amplification processes offer multiple advantages if compared with thermal cycling requiring methods, such as reduced needs for instrumental complexity, thermal control and power consumption, thus rendering their integration in microfluidic platforms significantly easier. In addition, the use of isothermal amplification techniques coupled with miniaturized devices for analysis ensure a higher percentage of specific amplification product obtained from the targeted DNA template and reduced amplification bias, enabling also accurate and high fidelity single-cell genome amplification in nanoliter volumes.
